# Lung ion-fluoroscopy Guided Hadron therapy: LIGHT concept and proof-of-principle

**DOI:** 10.1088/1361-6560/ae788c

**Published:** 2026-06-19

**Authors:** Saad Shaikh, Mikaël Simard, Ronja Hetzel, Maximilian Dick, Uli Weber, Kilian-Simon Baumann, Christian Graeff, Charles-Antoine Collins-Fekete, Lennart Volz

**Affiliations:** 1 Department of Medical Physics and Biomedical Engineering, University College London, London, United Kingdom; 2 Biophysics, GSI Helmholtzzentrum für Schwerionenforschung, Darmstadt, Germany; 3 University of Applied Sciences, Institute of Medical Physics and Radiation Protection, Giessen, Germany; 4 Department of Radiotherapy and Radiooncology, University Medical Center Giessen-Marburg, Marburg, Germany; 5 Marburg Ion-Beam Therapy Center (MIT), Marburg, Germany

**Keywords:** carbon therapy, real-time ion imaging, online treatment monitoring, plastic scintillator

## Abstract

*Objective.* This work provides proof-of-concept for the use of real-time ion imaging and treatment gating for lung cancer radiotherapy using 3D range modulators (3DRM). *Approach.* The accuracy of a fully real-time, plastic scintillator-based portal ion radiography detector was determined by tracking a 3 cm spherical plastic tumour undergoing breathing-style motion in a mock lung geometry. The ability to gate the delivered treatment using a trigger from the ion radiography detector at the desired tumour position was investigated. Finally, an offline simulated study was performed to compare the dosimetric benefits of the advanced image guidance against the standard clinical motion mitigation practice of rescanning. *Main Results.* The ion radiography detector was shown to track tumour motion in a mock lung phantom setup to 0.1 mm accuracy. The imaging dose was found to be approximately 1/3 of comparable x-ray fluoroscopy methods. While dynamically driving in the 3DRM is not currently possible, preliminary measurements showed modulator positioning to be reproducible to 0.3%. A technical demonstration was provided showing a real-time switch from imaging to treatment using a trigger from the ion-imaging detector, with the mock tumour found in the expected position. The simulated dosimetric study showed that the tracking accuracy in an idealised scenario allows the tumour to be treated quasi-statically, with a $D_{95\%}$ of 98% and 99.2% using passive and active treatment beam energy switching respectively. *Significance.* Lung Ion-fluoroscopy Guided Hadron Therapy (LIGHT) provides a promising approach for (a) mitigating tumour motion using a real-time, in-plane ion imaging device and (b) avoiding interplay effects with scanned beams by using a patient-specific 3D-range modulator to passively scatter a mono-energetic treatment beam. This technique also shows promise for future FLASH treatment delivery methods. Future work will fully determine the tracking performance and dosimetric benefits of LIGHT in more realistic clinical scenarios.

## 1. Introduction

Particle therapy offers improved dose localisation and therefore improved radiation induced toxicity when compared to conventional photon based radiotherapy (Paganetti 2017). However, due to the strong dependence of the dose deposition on the water-equivalent thickness (WET) path length, anatomical changes during and between treatment sessions can drastically alter the delivered treatment, resulting in under-dosing of the tumour and healthy tissue receiving increased dose (Lomax 2008, 2008b). In particular, treating lung cancers with particles presents significant challenges with anatomic heterogeneity and managing tumour motion caused by breathing, which significantly worsen dose conformity and can result in dose being delivered to the heart (Mori and Knopf 2018, Pakela *et al* 2022). Additionally, the modern standard for particle beam therapy delivery, pencil beam scanning, presents additional complications due to the interplay effect caused by scanning a moving tumour (Seco *et al* 2009). Unfortunately, it has been shown that this motion cannot be generally accounted for, as it is dependent on tumour location, volume, clinical stage and therefore requires patient-specific interventions.

Current methods of motion management for lung radiotherapy involve 4D computed tomography (CT), rescanning, gating, abdominal compression, deep inspiration breath-hold, implanted markers, fluoroscopy and surface tracking (Zhang 2023). Each of these techniques comes with certain benefits and setbacks: 4DCT-based treatment planning allows anticipation of motion induced dose errors during plan optimization (Knopf *et al* 2022), but is not yet available at isocentre and cannot be used for real-time guidance. Motion monitoring can be performed with abdominal compression belts or surface monitoring (Pakela *et al* 2022), but these techniques measure motion surrogates which generally do not correlate well with actual target motion. Internal imaging in the form of x-ray fluoroscopy for particle therapy has been investigated with promising results (Mori *et al* 2019). However, high imaging doses of ${\sim}24\,$mGy per image (about 0.5 Gy per treatment fraction (Mori 2016)) and the lack of information on the beam range present challenges for this method. Most commonly, beam rescanning and/or gating is applied to mitigate motion during delivery (Zhang 2023). Rescanning reduces the interplay effect between target and beam scanning motion, but cannot help to reduce the high dose volume. Gating crucially depends on accurate feedback on the tumour position and is subject to the residual motion during the gating window (Dolde *et al* 2019).

Particle radiography is an emerging technology that performs imaging using ion species such as protons and carbon. The main benefit over conventional photon-based imaging is the direct measurement of particle relative stopping power, thus eliminating the need for inexact conversion from CT Hounsfield unit, which is a large source of uncertainty in treatment planning (Parodi 2019). Integrated mode particle radiography (IMPR), wherein images are reconstructed by measuring low-dose, high-energy particle beams passing through a region of interest, is a promising method of providing minimally-invasive, $in$-$vivo$ beam characterisation (Gottschalk *et al* 2011, Rinaldi *et al* 2014, Tanaka *et al* 2016). While suffering from worse image quality and increased dose when compared to single-event particle imaging (Krah *et al* 2018), the far simpler technological requirements coupled with the ability to use standard clinical beams ideally positions IMPR for practical clinical applications. Recently, it has been shown that IMPR using a monolithic scintillator-based detector can offer competitive image accuracy when using advanced 2D reconstruction models (Simard *et al* 2024, 2025) as well as the capacity to track lung tumour motion (Fullarton *et al* 2025).

3D-printed 3D range modulators (3DRMs) have recently become of interest as a way to bypass slow treatment delivery times in most clinical particle therapy centres while still taking advantage of scanned beam delivery (Simeonov *et al* 2017, 2022). These modulators, typically made from polymer resin, can be designed on a patient-specific basis following treatment planning imaging and can provide highly conformal, spread-out Bragg peaks using a single mono-energetic scanned layer. With no energy-switching, treatment time is drastically reduced and notably, energy selection beam intensity losses are also avoided. As such, the application of these devices is of great interest to FLASH radiotherapy (Jolly *et al* 2020) and of relevance to this work, mitigating motion interplay effects in scanned particle therapy (Pakela *et al* 2022). Simeonov *et al* have recently demonstrated that a lung tumour carbon ion treatment field delivering 0.5 Gy can be delivered in just 0.2 s at a clinical carbon ion therapy centre (Simeonov *et al* 2022). In such a short delivery time the tumour can be treated as quasi-static. While this holds enormous potential for motion mitigated treatments, correct alignment of the 3DRM with the target is crucial, since the fast treatment prevents the use of motion mitigation techniques such as rescanning over different breathing phases. If the tumour is not well aligned with the planned position, healthy tissue receives the full treatment dose.

In this paper, an introduction and early proof-of-concept is provided for LIGHT: Lung Ion-fluoroscopy Guided Hadron Therapy. LIGHT uses advanced image guidance from integrated-IMPR with a high-energy, low-dose imaging beam to track tumour motion and trigger a dynamic 3DRM to move into the beam path, switching to a higher dose treatment beam to rapidly deliver the prescribed dose to the tumour in the desired position. In principle, this system can provide patient-specific monitoring of lung tumour motion, removing the need for intrusive or restrictive motion management interventions, and rapidly deliver treatment to mitigate interplay effects, reducing the need for time-intensive practices such as rescanning. This work builds on earlier work where proof-of-concept was provided for a scintillator-based integrated-mode proton radiography system to track objects in motion (Fullarton *et al* 2025). Here, an improved system is presented that provides fully real-time motion tracking in a more compact footprint and with simpler image processing, along with a simulated dosimetric study of the improvements provided by the LIGHT setup.

## 2. Ion imaging Guided 3DRM treatment

**Figure 1.**
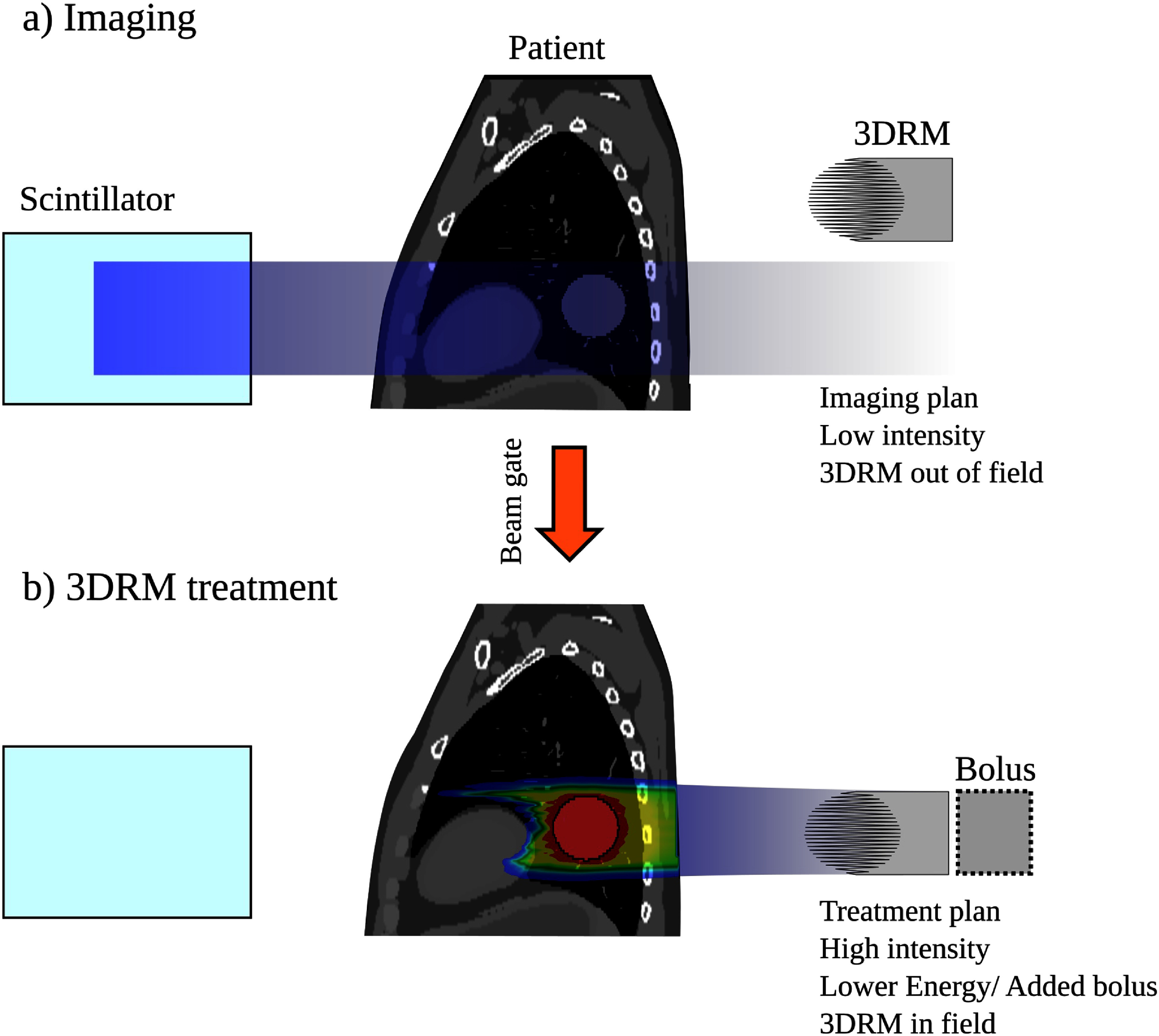
Schematic overview of the LIGHT concept: (a) prior to treatment, the 3D range modulator (3DRM) and bolus (if any) are moved out of the treatment field. The beam passes through the patient and permits dynamic ion radiographs. (b) As soon as the radiographs indicate the tumour to be in the planned treatment position, the beam is briefly gated, while the 3DRM (and bolus) are moved into the beam. The beam intensity is increased to clinical levels, and the treatment plan is irradiated.

The core idea of LIGHT is shown in figure 1: at the start of treatment, the 3DRM is positioned out of the treatment field, on a fast linear stage. The treatment commences with low dose ion imaging, for which a high beam energy and low beam intensity is used. When the ion fluoroscopy indicates the tumour to be in the right position, a switch to the treatment field is triggered. The beam is gated, while the 3DRM is moved into the treatment field, and the beam energy is decreased, either by multi-energy extraction (Yap *et al* 2021) or via a bolus that moves into the beam together with the 3DRM. As soon as the passive devices are in place, the beam gate is opened, and the beam intensity is increased to the clinical levels, in order to deliver the treatment field. The treatment is then applied as in (Simeonov *et al* 2022). As the ion imaging is low dose, it only requires a short percentage of the synchrotron spill, with only small impact on the maximal dose that can be delivered in a single spill. In addition, by monitoring the tumour with the beam itself, registration/transformation are kept minimal. Finally, the ion radiographs also provide information on the patient water equivalent thickness, possibly permitting monitoring not only of the lateral tumour position, but also any beam range changes. Using available technology, driving in the passive devices into the beam can be done in order of one to two hundred milliseconds, assuming the out-of-field position to be 20 cm from the isocentre, and a triangular acceleration profile of 10–20 m s$ ^{-2}$. To demonstrate this concept, three things need to be proven:

• Ion fluoroscopy can provide tumour alignment feedback in real time
• The 3DRM can be moved quickly into the treatment field, with acceptable (i.e. smaller than 1%) loss in dosimetric accuracy
• The treatment delivery system can switch quickly from imaging to treatment mode

This work aims to provide an early proof-of-concept for these points, using available technology from GSI and UCL for experiments at Marburger Ionenstrahl-Therapiezentrum (MIT), one of the two German carbon ion therapy centres and at the research centre GSI where the German pioneer carbon ion treatments where performed.

## 3. Materials and methods

This work was conducted in 3 separate stages to evaluate the key aspects of the proposed LIGHT setup:

(i) Motion tracking: experiment at MIT to evaluate the accuracy of a real-time integrated IMPR detector in tracking periodic tumour motion.
(ii) Treatment gating: experiment at GSI to demonstrate real-time imaging to treatment triggering using ion fluoroscopy.
(iii) Dosimetric evaluation: analytical simulation study to evaluate the potential dosimetric benefits of the LIGHT setup compared to standard clinical practice.

### 3.1. Motion tracking

#### 3.1.1. Ion imaging detector

**Figure 2.**
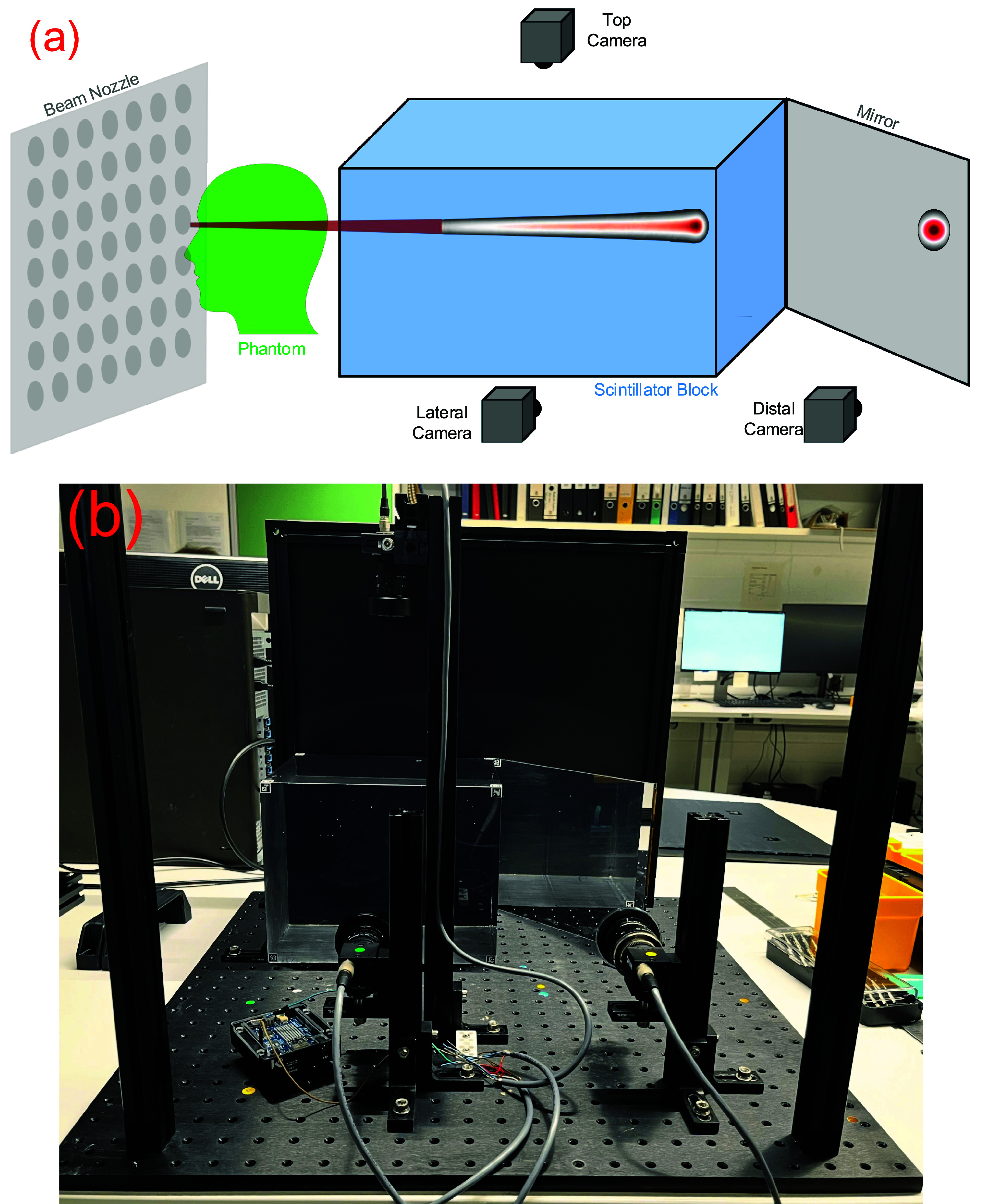
(a) Simplified detector schematic showing detection principle and (b) assembled detector in enclosure (without light-shielding foam boards).

The detector design motivation, justification and use-cases have been discussed in previous works (Tendler *et al* 2021, Darne *et al* 2022, Simard *et al* 2024). The detector is a NUVIATech Instruments monolithic polystyrene scintillator block of lateral surface area 20 $\times$ 20 cm and depth 25 cm. The scintillator has peak emission at 425 nm, refractive index of 1.57, density of 1.03 g cm$ ^{-3}$, light output 56% of anthracene and decay constant of 2.5 ns (NUVIATech Instruments 2019). The block is imaged from three angles using three compact FLIR BFS-U3-04S2M-CS USB3 cameras (FLIR Integrated Imaging Solutions 2018), as shown in figure 2(a).

The camera captures full-frame images at up to 522 Hz and uses a Sony IMX287 1/2.9” CMOS imaging sensor, which provides a 0.4 MP 720 $\times$ 540 pixel image resolution. Only images captured from the beams-eye view camera, referred to as the distal view, are used in this work. This view used a Kowa LM6HC 6 mm fixed focal length lens (Kowa Optimed 2019) to image the rear scintillator face through an Edmund Optics 4–6$\lambda$ mirror of size 200 $\times$ 235 mm (Edmund Optics 2025) placed at 45$ ^{\circ}$ to the scintillator face. This was to protect the camera from stray neutron radiation dose beyond the Bragg peak and resulted in a camera-mirror distance of approximately 30 cm. The detector was housed in a ThorLabs optical breadboard enclosure to provide light-shielding from the surroundings, which was 52.5 cm tall and had a footprint of less than 60 $\times$ 60 cm in area. The assembled detector is shown in figure 2(b).

To synchronise image capture with the particle beam delivery system, two diagnostic signals from the accelerator were used: the beam magnet position and the magnet switching signal. These were used as proxies for ‘spill-on’ and ‘next-spot’ signals respectively to start camera exposure, keep track of the spot number, and then stop/restart exposure for the next field delivery. An Arduino UNO R4 WiFi (Arduino 2025) was used to perform logic with incoming accelerator signals and output square-wave trigger signals to the cameras via 6-pin Hirose cables. All cameras were connected via USB3 to an ASUS NUC 14 Pro mini PC (Intel 2024) for data acquisition. Custom C++ code using the Spinnaker SDK (FLIR Integrated Imaging Solutions^2025^) was written to configure camera parameters and acquire images.

#### 3.1.2. Motion platform

**Figure 3.**
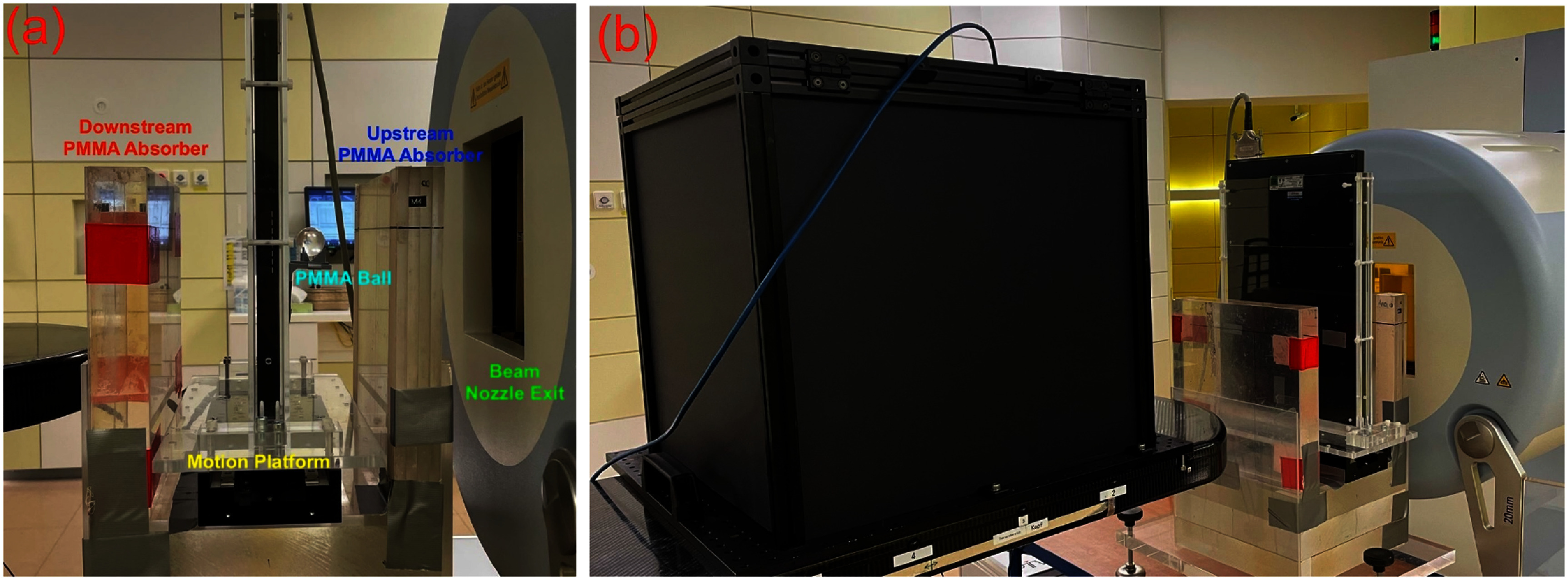
Experimental setup at MIT showing (a) close-up of dynamic phantom and (b) detector in enclosure in beamline.

Radiographs were captured of a 3 cm diameter PMMA ball phantom in motion, which was held on a motion platform placed approximately 19 cm from the beam nozzle, around 50 cm upstream of the particle radiography detector. The front of the scintillator block was placed at isocentre. Additionally, 52.83 mm WET of PMMA absorber was placed upstream of the motion platform and 46.35 mm WET downstream to provide a more patient-realistic geometry for the imaging use-case. The experimental setup is shown in figure 3.

The ball underwent an asymmetric sinusoidal motion (perpendicular to the beam direction) to mimic the inhale-exhale lung breathing pattern (Lujan *et al* 1999), where the relatively long, stable ‘exhale’ state was chosen as the phase to deliver treatment. A total of 9 different motion patterns were tested, corresponding to amplitudes of 10 mm, 20 mm and 30 mm and time periods of 2 s, 3 s and 4 s. The platform itself was a precision linear stage (Model M404.2PD, Physik Instrumente, Karlsruhe, GER), which executed the preprogrammed breathing motion. For a reproducible setup, the motion was triggered via the “beam on” trigger signal from the accelerator.

#### 3.1.3. Imaging Field & Dose

In this experiment, images of a 6 cm $\times$ 5 cm 344.6 MeV/u carbon ion field were captured. The beam width at isocentre was 6 mm FWHM. It was chosen to deliver spots with 5 mm spacing as a compromise between imaging speed and quality (Fullarton *et al* 2025). As such, each field contained 143 spots of 100k particles and took approximately 195 ms to deliver, thus providing a 5 Hz imaging frame rate. Dose calculations were performed with TRiP98 (Krämer *et al* 2000) on a water phantom, yielding an average dose of ${\sim}8\,$mGy per 1 mm$^3$ voxel in the central part of the imaging field.

#### 3.1.4. Image reconstruction

OpenCV libraries (Bradski 2000) were used to perform image processing and reconstruct the position of the phantom in real-time as images were acquired from the camera. To facilitate true real-time imaging, a faster, simpler image processing method using only images from the distal view camera was used. The basic principle for image reconstruction is to subtract the image of an object from the image of the field without any object in its path, which should provide an image of the PMMA ball phantom in motion. A single image of the box field was captured and used as the background subtraction image for all subsequent runs. After subtraction, standard image correction protocols were performed (Robertson *et al* 2013), which were applied sequentially: spatial median filtering, vignetting correction and lens distortion correction. Otsu’s algorithm was then applied for image thresholding (Otsu 1979), after which contour detection was used to deliver the reconstructed phantom shape and centroid.

### 3.2. Treatment gating

Since the setup at MIT is a medical product, it has limited flexibility for investigating new treatment delivery concepts. For a qualitative proof of concept to dynamically switch between the imaging and treatment plan, the experiments performed at MIT were replicated at the GSI medical research room, ‘Cave M’ (Lis *et al* 2021). The GSI dose delivery system (DDS) is a research version of the clinical DDS at Centro Nazionale di Adroterapia Oncologica (CNAO, Pavia, IT). The DDS has already been extended to deliver a library of plans for the case of motion synchronized beam delivery (Steinsberger *et al* 2023). For motion synchronized deliveries, the DDS expects the current breathing phase from a motion monitoring system. It then automatically selects the right plan for that motion phase from a library of treatment plans, where one plan is available per motion phase (typically ten phases in total). Here, this infrastructure was exploited to deliver a long chain of imaging fields concatenated into a single plan for the ‘imaging phase’ (phase 0), and the treatment plan itself as a ‘treatment phase’ (phase 1). During the imaging phase, the acquired scintillator images for each imaging field were evaluated as soon as the exposure ended, to produce a real-time tumour centre-of-mass location. When the tumour centre-of-mass position was within 1 mm of the isocentre position, the phase 1 signal was given to the DDS to trigger the treatment via a routine implemented on an Arduino UNO R4 WiFi.

The setup at GSI was the same as used for the experiments at MIT. For imaging, the same plan geometry as used at MIT were applied, but the beam energy was 240 MeV u^−1^. At this stage, driving in the modulator quickly enough to support the dynamic treatment was not yet possible at GSI. For the proof of concept, a single-energy-layer 240 MeV u^−1^ plan was chosen to demonstrate the dynamic switch from imaging to treatment mode. The treatment plan was a circular irradiation field of 18 mm radius, enclosing the PMMA ball with a 3 mm isotropic safety margin. Each spot comprised 2 million carbon ions, and spot spacing was $2\times 2\,$mm.

### 3.3. Dosimetric evaluation

Online tracking results were used in an offline simulated dosimetry study to evaluate the dosimetric improvements provided by the advanced image guidance from the ion radiography detector. Treatment plans were created with GSI’s in-house research treatment planning system for ion beam therapy, TRiP98 (Krämer *et al* 2000). First used for treating patients during the GSI carbon ion therapy pilot project between 1997 and 2008, TRiP98 has been continuously upgraded to enable advanced treatment planning features, such as 4D optimization, robust optimization (Wolf *et al* 2020), ion arcs (Volz *et al* 2024), and range shifter deliveries.

Single-field carbon ion treatment plans were generated for an XCAT digital patient (Segars *et al* 2010), featuring a 5 cm diameter spherical tumour in the left lobe of the lung. To simulate anatomical motion, 25 XCAT CTs were generated, each corresponding to an $n$ times 1.5 mm linear displacement of the diaphragm and tumour. From these 25 CTs, a 4DCT of 10 phases was sampled according to a breathing motion with 20 mm amplitude (min-to-max), following the methods presented in Steinsberger *et al* (2023). To assess the possible benefit of the 3DRM treatment, four scenarios were compared:

(i) Ideal clinical scenario: 4D internal target volume (ITV) (Steinsberger 2025) treatment plan, perfect rescanning (i.e. no interplay effect).
(ii) Realistic clinical scenario: range ITV plan, with dose calculated using MIT delivery parameters and 4 rescans. No additional motion mitigation is assumed.
(iii) 3DRM (low energy): quasi-static 3DRM treatment with active switching between imaging and treatment beam energy.
(iv) 3DRM (high energy): same as (iii) but with passive energy switching using an added bolus.

In the treatment planning and dose calculation, the 3DRM was approximated as a range shifter comprising a set of eight discrete slabs of material with water equivalent thicknesses between 0.25 mm and 64 mm in steps of powers of two. For the low energy 3DRM plan, the beam range was chosen according to the maximum range of the target, and all other range changes where done by adding a combination of the range shifter material. For the high energy 3DRM plan, the beam range was set to 350 MeV/u, which was sufficient to cross the patient completely. A fixed bolus of 120 mm water equivalent thickness was then added, to pull the range back to the treatment range. Further range changes were computed with the discrete range shifter as for the low energy 3DRM plan. Except for scenario (ii), plans were optimized with a minimum spot weight of 15k carbon ions/spot. To enable 4 times rescanning, the minimum particle limit was increased by a factor of 4 for scenario (ii). For this scenario, a 4D-dose was computed with TRiP98, simulating treatment delivery according to the accelerator setup at MIT, using the GSI in-house beam delivery simulator (Richter *et al* 2013).

Plans were robustly optimized for a single fraction dose of 3 Gy(RBE) on the CTV, assuming 3 mm setup and 3% range uncertainty. For biological dose calculation, LEMIV (Pfuhl *et al* 2022) was used with an $\alpha/\beta$ ratio of 6 in the tumour, and 2 elsewhere. The left lung and heart were set as organs-at-risk during optimization. Spot spacing was $2\times 2$ mm, and iso-energy layers were spaced 3 mm. Plans were evaluated for the target coverage, $D_{95\%}$ and $V_{95\%}$. For evaluating the dose to organs at risk, $V_{30\%}$ was used as metric for the lung, while $D_{\mathrm{max}}$ and $D_{\mathrm{mean}}$ were used for evaluating the heart dose.

## 4. Results

### 4.1. Motion tracking

**Figure 4.**
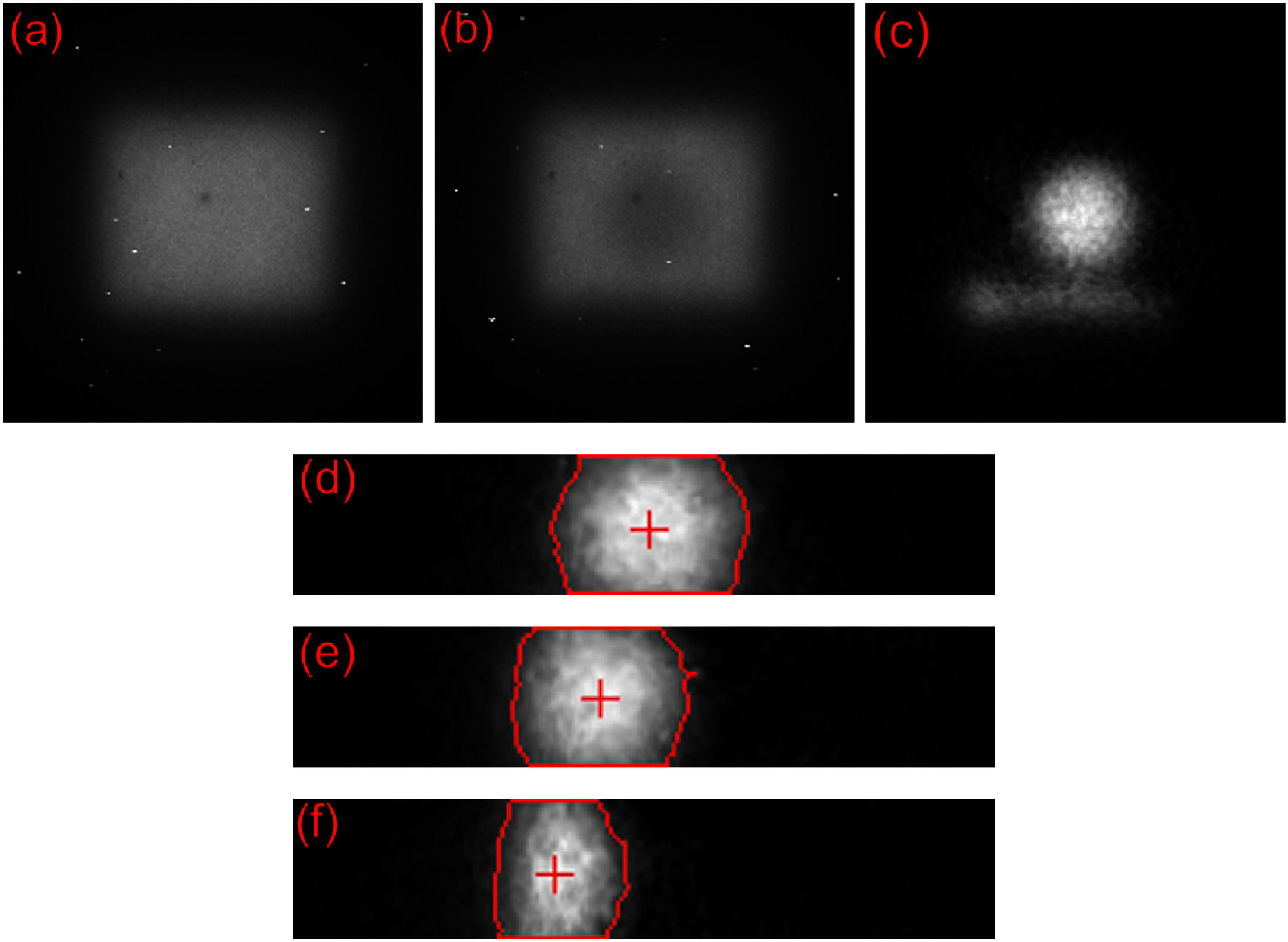
(a) Raw background image of 6 cm $\times$ 5 cm 344.6 MeV/u carbon ion box field, (b) raw image of field with PMMA ball phantom placed in beam path, (c) reconstructed image showing ball and mount and (d)–(f) cropped image with tracking contour and centroid for maximum displacements of 10 mm, 20 mm and 30 mm amplitude patterns respectively.

Raw background and signal images taken by the radiography detector are shown in figures 4(a) and (b) respectively. The resulting image after performing the image correction and reconstruction process outlined in section 3.1.4 is shown in figure 4(c). Figures 4(d)–(f) shows the cropped images with tracking results, showing the detected contour and centroid, at the maximum displacement for each motion amplitude. A 1$ ^{\circ}$ rotation was observed in the images due to slight misalignment of the mirror, which was corrected for during image processing. The computation time for image corrections and object tracking was found to be around 8 ms per image, negligible to the 195 ms taken to deliver the field and therefore providing the maximum 5 Hz imaging frame rate.

It can be seen that the width of the tracking contour at maximum displacement shown in shrinks with increasing amplitude of motion. This is due to a motion artefact from the 0.2 s imaging time, which was shown in previous work (Fullarton *et al* 2025) to distort the shape of objects, especially during directional changes in motion. Additionally, for the 30 mm amplitude case in figure 4(f), the ball moves partially out of view of the imaging field. This behaviour is acceptable for the proposed use-case, as it is only necessary to be able to detect when the object is in the stable exhale phase (in this case, at 0 mm displacement). Furthermore, restricting the imaging field to only the treatment window would limit the dose delivered to healthy tissue.

**Figure 5.**
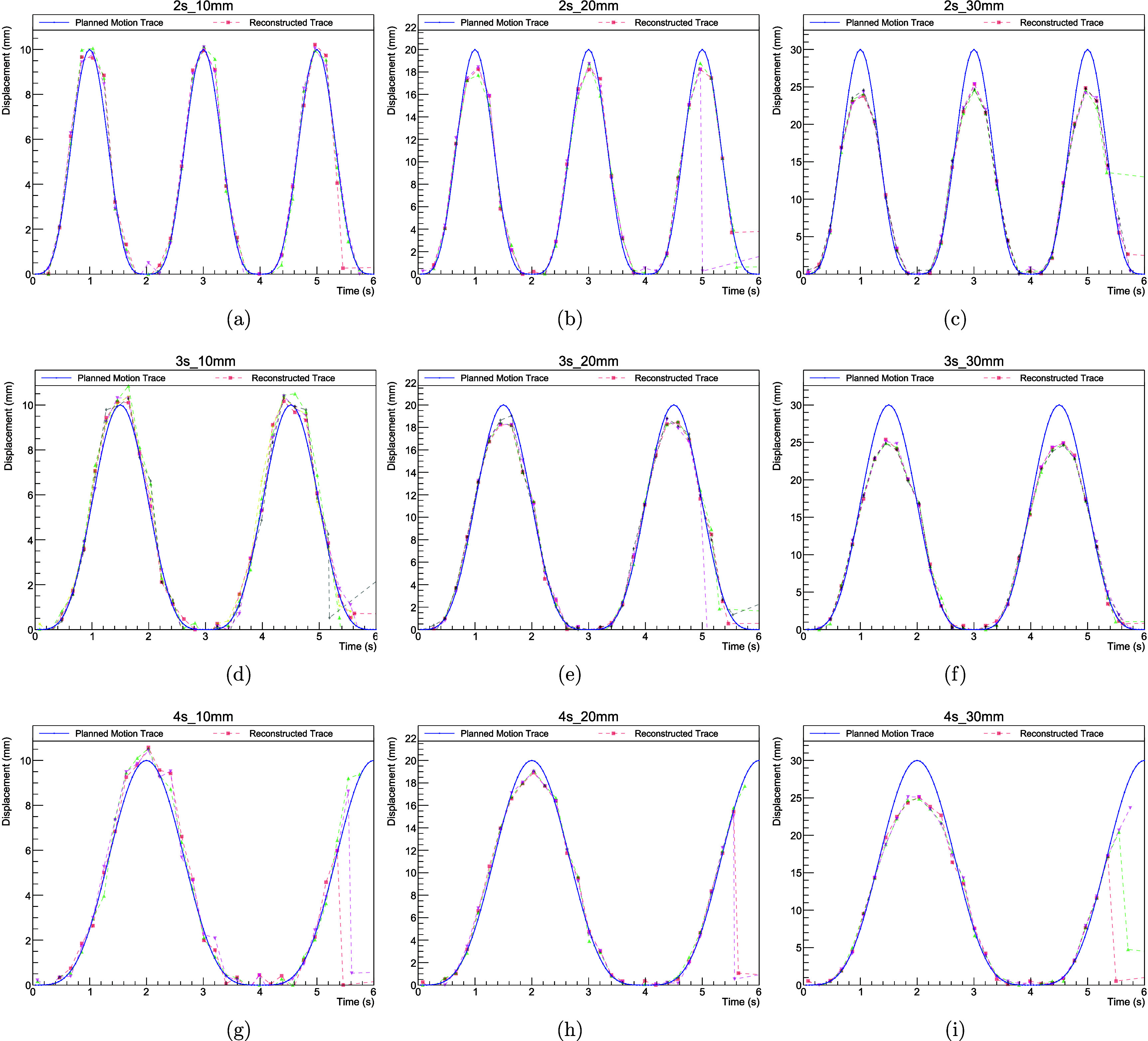
Planned and reconstructed motion traces for (a) 2 s period, 10 mm amplitude, (b) 2 s period, 20 mm amplitude, (c) 2 s period, 30 mm amplitude, (d) 3 s period, 10 mm amplitude, (e) 3 s period, 20 mm amplitude, (f) 3 s period, 30 mm amplitude, (g) 4 s period, 10 mm amplitude, (h) 4 s period, 20 mm amplitude and (i) 4 s period, 30 mm amplitude. The image timestamps were shifted by a constant value of 0.08 s to align with the clock of the motion platform.

After correcting images for optical effects, the pixel number for the tracking centroid was converted to physical position, taking into account for perspective effects from the depth of the treatment plan (see appendix A.2), which gave a physical pixel size of 0.529 mm. The planned motion and detector reconstructed traces for each motion pattern are shown in figure 5. For all motion patterns, a good match can be observed between the planned and reconstructed trace. The drop in reconstructed peak height for the 20 mm and 30 mm amplitude patterns are attributed to the aforementioned motion artefacts and limited imaging window. To quantitatively evaluate tracking accuracy for each pattern, the average difference in ball displacement was calculated for each acquired image, for displacements under 15 mm, to avoid the peaks where motion artefacts were present. The results are summarised in table 1. Taking a simple average for all patterns gives an overall position accuracy of 0.1 mm, well within the 1 mm resolution relevant for treatment planning systems.

**Table 1.** Reconstructed trace accuracy (mm) for each motion pattern for displacements less than or equal to 15 mm.

	Amplitude (mm)		
Time period (s)	2	3	4
10	0.122	0.184	0.146
20	0.076	0.025	0.093
30	0.046	0.033	0.183

### 4.2. Treatment gating

**Figure 6.**
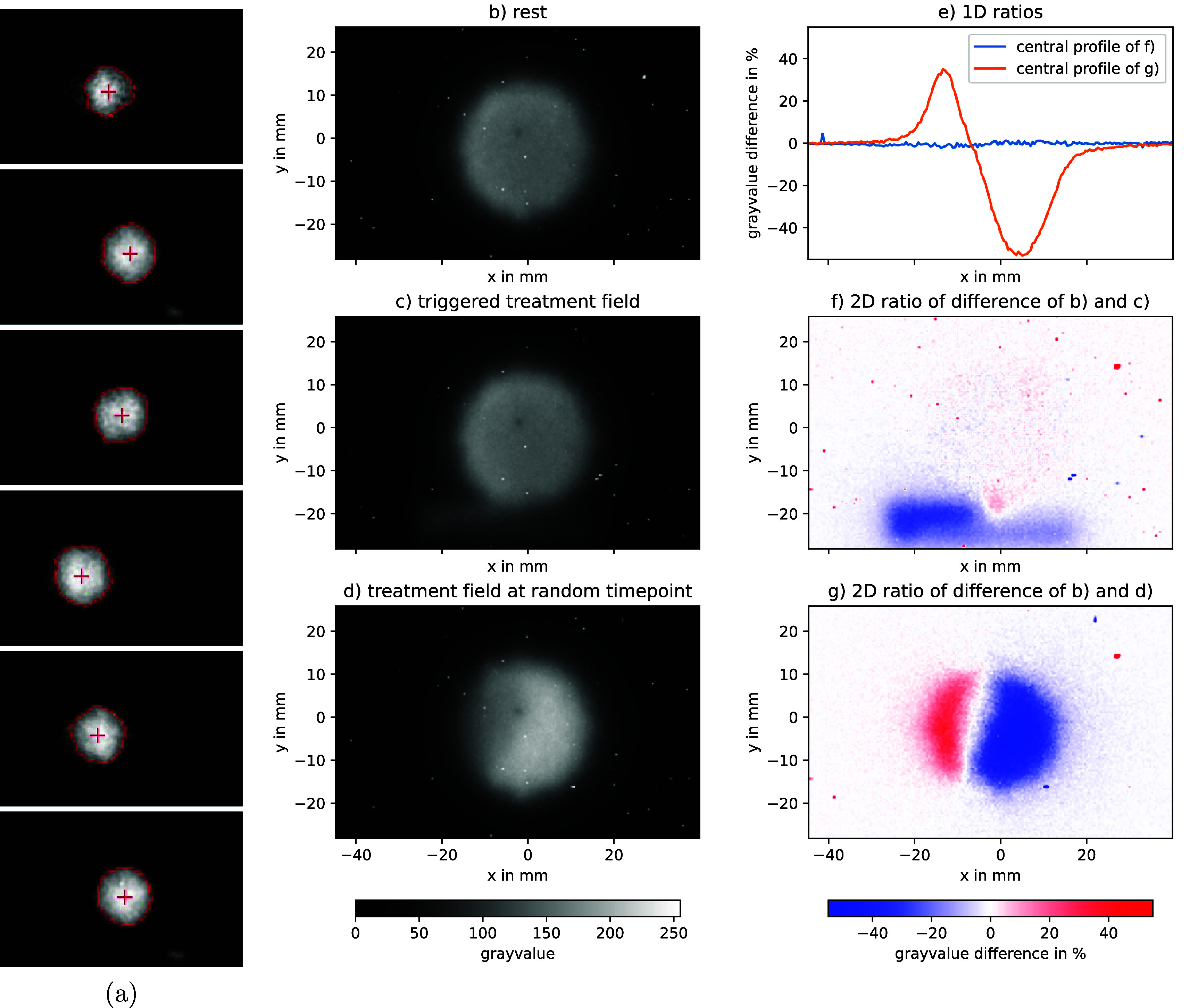
Series of 6 reconstructed images of the PMMA ball in motion acquired at GSI, showing tumour contour and centroid position in (a), and comparison of images taken of the treatment field with the ball: (b) in rest, (c) in motion with ion fluoroscopy triggering and (d) at a random time point during motion. Figures (f) and (g) show the ratios of the images in motion to the image in rest and in figure (e) a 1D projection of the 2D ratios in the central region is displayed. In (b) part of the last imaging field is still visible in the image of the treatment field creating the blue area in (e).

Figure 6 shows a series of images captured by the rear-view camera during the ‘imaging phase’ and a comparison between the central profile across the scintillation light output for the dynamically triggered treatment field and a static treatment, where the ball was manually positioned in isocentre via the linear stage. Since the beam energy was not reduced between imaging and treatment, the treatment plan also stopped inside the detector, permitting evaluation of the capture quality. The two profiles agree well with each other, qualitatively proving the possibility to trigger a treatment based on the real-time scintillator feedback.

While it was not yet possible to move in the 3DRM into the treatment field at the desired speed, due to unavailability of suitable equipment, a test was performed with a pneumatically driven target shifter at MIT. The depth-dose profiles measured for the 3DRM after multiple automatic repositioning runs are provided in the appendix, figure 8(b). The 3DRM was moved 25 cm in ${\sim}1$ s. The mean difference between dose profiles after repositioning compared to the reference was 0.3%.

### 4.3. Dosimetric evaluation

**Figure 7.**
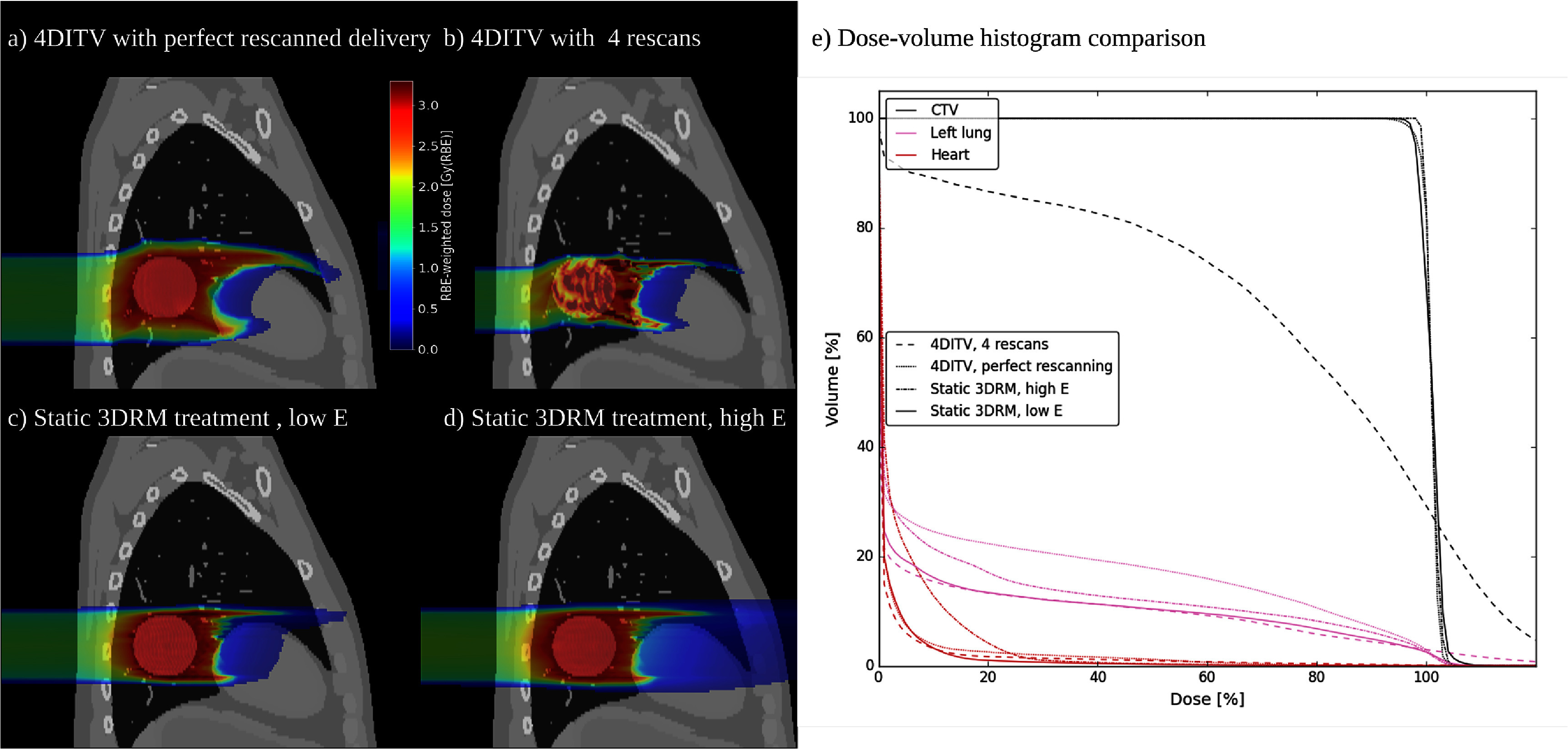
Dosimetric analysis of the benefit of a static 3DRM treatment versus an ITV treatment. (a)–(d): Comparison of dose slices overlaid on the XCAT CT reference phase. (a) and (b) were computed on the full 4DCT of the patient with perfect and realistic rescanning respectively. (c) and (d) are static 3DRM treatments on the reference phase assuming active and passive energy switching respectively. In active switching, the beam energy was chosen to be equal to the maximum range of the target whereas for passive, the beam energy was changed with a 120 mm WET bolus in front of the patient, with the beam energy kept at 350 MeV/u.

For the most representative motion pattern of lung breathing motion (4 s time period, 20 mm amplitude (Seco *et al* 2009)), the treatment trigger time was chosen as the first tracking result within 1 mm of the exhale minimum phase. Additionally, the effect of a finite time for trigger signals to propagate, motion platform to move into place and the treatment beam to switch on must also be considered. For the purposes of the simulated dosimetry study, several different time scales were considered: 1 ms, 10 ms, 100 ms and 1 s. For three repeats of the 4 s time period, 20 mm amplitude motion, the average true ball position for the above timescales are respectively: 0.84 mm, 0.80 mm, 0.43 mm and 0.18 mm. The delivery of a lung 3DRM plan in a single-spill has been shown to be on the order of 200 ms at MIT (Simeonov *et al* 2022). This would mean the tumour would remain closer than 1 mm to the reference position during treatment delivery for all switching times presented above for this motion scenario. Since this is smaller than the voxel size of the digital XCAT phantom, and much smaller than the voxel size for typical simulation CTs used clinically, it is reasonable to assume a static delivery of the 3DRM plan in the reference phase (end-exhale). Hence, in the dosimetric comparison, the 3DRM plans are static dose calculations on the reference CT. The results are shown in figure 7. Quantitative DVH metrics are provided in table 2.

**Table 2.** DVH metrics for the different plans shown in figure 7.

Plan	4DITV, perfect rescanning	4DITV, 4-times rescanned	3DRM, high energy	3DRM, low energy
CTV, $D_{95\%}$	$98.5$%	$0.7$%	$99.2$%	$98.0$%
CTV, $V_{95\%}$	$98.5$%	$37.1$%	$100.0$%	$99.9$%
Lung, $V_{30\%}$	$12.2$%	$20.7$%	$14.2$%	$12.1$%
Heart, $D_{\mathrm{max}}$	$99.4$%	$150.3$%	$100.9$%	$104.2$%
Heart, $D_{\mathrm{mean}}$	$2.0$%	$1.6$%	$3.7$%	$1.4$%

Both static 3DRM deliveries provide superior plan quality when compared to the clinically realistic beam delivery scenario. The low energy 3DRM provides similar plan quality to the ideal 4DITV case. The high energy 3DRM plan has slightly higher mean heart dose and elevated low dose to the heart due to the additional carbon ion fragments from the bolus in the beam path. It is noted that all scenarios could be improved further with the addition of non-fluoroscopic motion tracking techniques, but with no suitable system available at GSI or MIT at the time of the experimental campaign, this was beyond the scope of this work.

## 5. Discussion

### 5.1. Detector performance

The sub-mm tracking accuracy demonstrated in this work is well within the 1 mm resolution relevant for treatment planning, however this is expected to worsen in more complex geometries with smaller, non-uniformly sized tumours and with motion non-perpendicular to the beam direction. Future work will therefore continue stress-testing the beam’s-eye view approach in more realistic heterogenous geometries, e.g. with the Sun Nuclear dynamic thorax phantom (Sun Nuclear 2025). As the resulting image captured in a more realistic scenario would be considerably more complex, methods to ensure adequate target delineation will need to be determined. One approach could be to incorporate WET sensitivity by calibrating the light intensity from the distal view camera (Olivari *et al* 2025) or from range information captured by the lateral view cameras (Simard *et al* 2024).

Motion artefact interplay effects were observed in the reconstructed images, which were expected due to the scanned beam delivery system. While not problematic in detecting the phantom in the exhale phase, the sampling resolution can be improved by reducing the time taken to deliver the field, reducing the size of the field and reducing the spot spacing. Nevertheless, a sampling rate of 5 Hz appears to be adequate for tracking the motion of a typical breathing cycle and reducing the spot spacing further would worsen the overall image quality. The exact imaging field size is expected to vary on a case-by-case basis depending on initial exploratory imaging to examine the patient-specific breathing motion pattern and optimal treatment position. Additionally, this experiment was performed with carbon ions, whereas protons are far more widely used for particle therapy. It has shown that carbon ions offer improved resolution when compared to protons due to less multiple coulomb scattering with carbon (Simard *et al* 2025), however it was noted that image quality with integrated-mode proton radiography is expected to remain acceptable for most use-cases.

In principle, image quality and resolution could be improved by capturing individual beam spots and summing to reconstruct the image of the field. However, while the cameras were in principle able to keep up with the beam scanning speed, it was found that controlling the camera exposure time using the next spot triggers (i.e. dynamically setting the exposure time to the spot dwell time), resulted in around 2 ms of deadtime between images. In this case, the camera must read out each line before the next image can be captured. This deadtime does not occur when setting a fixed camera exposure time, where the shortest exposure time (1.75 ms) could be used to oversample the scanned beam. However, this has the drawback of substantially increasing the image processing overhead and so for fully real-time performance, it was most appropriate to image the entire field. Future work will continue investigating the utility of fixed-time imaging for real-time applications.

Finally, as discussed in section 4.1 and appendix A.2, a beam energy-specific correction was applied to obtain an accurate pixel size in millimetres. While straightforward, a robust system should ideally have a consistent pixel size with beam energy. This unfortunately presents a practical compromise: increasing the focal length of the lens reduces this perspective magnification effect, however this then requires the camera-mirror distance to be increased, thus increasing the overall size of the detector. It is estimated that using a focal length lens of around 10 mm should sufficiently reduce this effect to not require a correction, at the expense of increasing the camera-mirror distance to around 50 cm. While the experimental setup uses three cameras, if the desired use-case only requires beam’s eye view imaging with the distal camera, then this increase in distance in favour of more robust imaging may very well be acceptable. Alternatively, an automated method using either the lateral or top camera view could be implemented to detect the beam position and calculate this correction when capturing the background empty field image. In contrast, flat-panel proton radiography approaches have been proposed in literature (Oria *et al* 2023), but these require lengthy, (patient-specific) energy calibration procedures to optimise the number of energy layers used for imaging. While benefiting from a smaller device footprint, the requirement for multiple energy layers (due to not capturing the full Bragg peak), results in larger imaging doses and acquisition times, thus making real-time motion tracking challenging.

### 5.2. Clinical applications

The LIGHT setup is designed to offer advanced image guidance to track lung tumour motion caused by breathing and mitigate slow scanning beam delivery times through 3D range modulation. In principle, this setup offers a non-invasive, patient-specific solution that removes the need to restrict patient breathing through mechanical or breath-hold techniques. Additionally, up-to-date information on inter-fractional anatomical changes to tumour size and shape can also be provided. Notably, the imaging technique used in this work provides direct in-plane imaging of the treatment site immediately prior to treatment delivery using the treatment beam, which is not possible with x-ray fluoroscopy or surface monitoring. At present, however, LIGHT can only monitor motion perpendicular to the beam, as discussed in section 5.1. Incorporating the system would be straightforward in horizontal beam lines (i.e. most currently operational carbon ion centres and future upright radiotherapy systems (Volz *et al* 2022)) or in gantry-based scenarios when treating from either 90$ ^{\circ}$ or 270$ ^{\circ}$ gantry angles, however full integration into a gantry system to enable monitoring at every gantry angle would require additional apparatus to rotate the detector around the patient.

LIGHT offers some possible advantages over x-ray fluoroscopy tracking systems by providing true beams-eye-view imaging, low dose, and the possibilities for detection of WET changes. The dose per image for the ion radiographs presented here is one third of what has been reported in comparable work with x-ray fluoroscopy (Mori 2016). It is important to point out that further dose reduction may be achieved with more sensitive detector equipment, possibly permitting even lower doses per image in the future. It is also noted that the low dose for imaging would also not reduce the available total number of particles per spill by much, and therefore, would not influence the delivery of the 3DRM dose in a single spill. However, it needs to be acknowledged that x-ray fluoroscopy has been a clinically mature modality for a decade at particle therapy centres (Mori 2016). Ultimately, the low imaging dose of ion imaging may make it possible to combine LIGHT with other established techniques to generate complementary information. This may also be interesting in guided breath-hold treatments, where a single ion radiograph right before the treatment delivery could serve as the final check of the tumour position. It is the authors’ intention to spark a discussion in the community on the integration of ion fluoroscopy for treatment guidance, rather than proposing to fully replace existing techniques.

As shown in figure 7, there are dosimetric benefits from actively switching the imaging beam energy for treatment, rather than passively scattering the beam using a bolus. The energy layer switching time for beam delivery systems can vary from tens of milliseconds to several seconds depending on the acceleration system used (Yap *et al* 2021). At least for the typical breathing pattern and with a 1 mm trigger threshold, this time delay should not prevent the use of this setup with most systems in operation today. It also is not anticipated that there would be marked differences in performance when comparing usage with cyclotrons, synchrotrons or synchrocyclotrons, or between raster-, spot- or line-scanning systems, as images of entire fields are integrated. Nevertheless, systems that can offer faster transverse scanning times will suffer fewer motion interplay effects. One exception to this is the spill structure of synchrotrons, which will cause a gap in imaging of a few seconds and thus increase the overall imaging time. This may be problematic in scenarios where the breathing cycle is synchronized with the spill, i.e. if the reference position is always reached during a spill pause. This would however still be detected with ion radiography and the patient could be advised to adjust their breathing.

**Figure 8.**
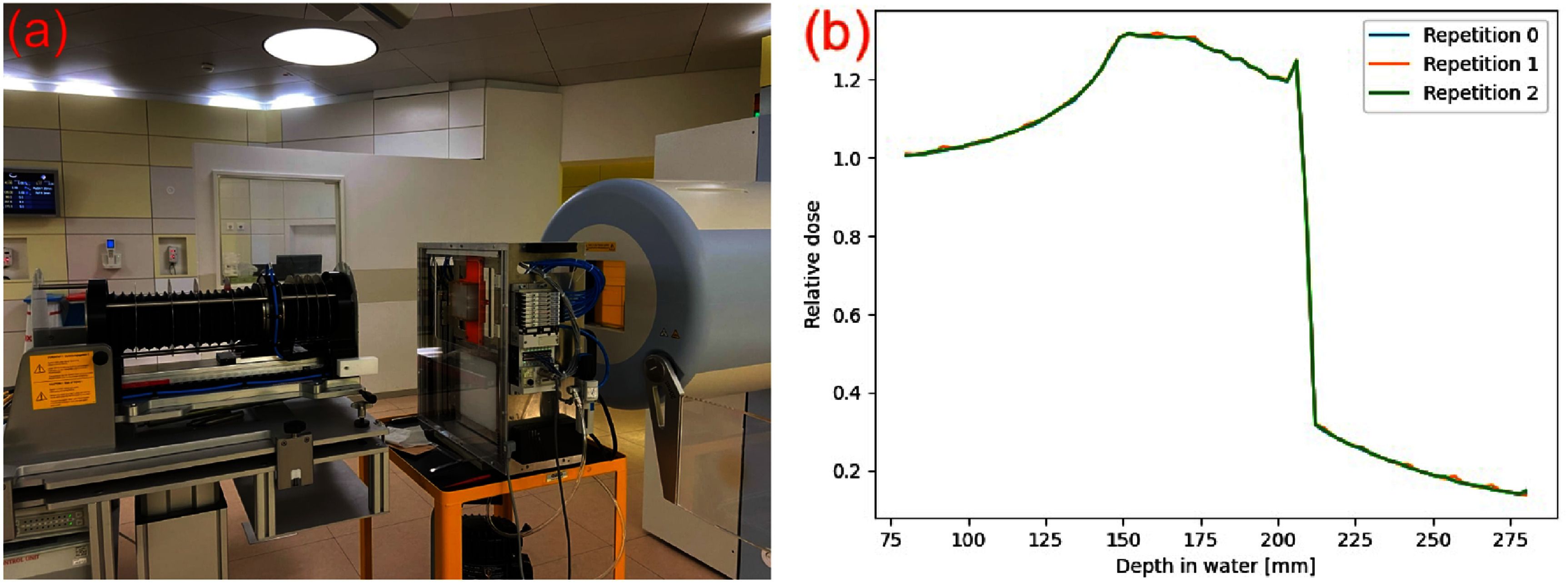
Comparison of the reproducibility of automatic 3DRM positioning with a pneumatic target shifter. (a) Setup at MIT, comprising the 3DRM mounted on the target shifter and the PTW PeakFinder. (b) Comparison of the depth dose profiles acquired for multiple repetitions, where repetition 0 is the reference profile.

While the full demonstration of LIGHT, including driving in the 3DRM at the desired speed, remains subject for future work, the potential to dynamically switch between an imaging and a treatment plan was shown at GSI. The qualitative results show the great potential of LIGHT: the dynamically triggered treatment matches the static, manually aligned treatment accurately. To achieve this, only the existing infrastructure for 4D-synchronized treatment delivery was used, which has already been extensively tested at CNAO (Steinsberger *et al* 2023). Since no further changes to the DDS would be needed, swift translation of LIGHT to patients would be possible in the future. Preliminary tests performed at MIT during the LIGHT experimental campaign indicated that the 3DRM can move 25 cm in $\sim1\,$s, without loss in dosimetric accuracy (see appendix and figure 8). Commercial options for moving the 3DRM with up to 5 g acceleration exist, but it needs to be investigated, whether the fast motion of the 3DRM would affect the treatment in any way. In the worst-case scenario at present, in which a trigger is given at the end of the end-exhale phase, where the tumour is moving fastest, it is estimated that the tumour could move a maximum of 2.6 mm in the ${\sim}$200 ms it takes for the 3DRM to move into place (for a fast 3 s, 20 mm breathing pattern). This remains within typical robustness margins of 3 mm/3%.

A simpler alternative to driving in the 3DRM at speed is to utilise a static 3DRM that is placed in the beam path during imaging, such that the 3DRM itself forms part of the background image. In principle, it should be possible to take this in account during the reconstruction process, however imaging quality may worsen due to the additional particle scatter. This will be investigated in future work with realistic 3DRM geometries. Nevertheless, for realistic clinical implementation, this presents a viable solution if the shock vibrations and/or electromagnetic interference from the 3DRM shifter proves too problematic for the beam nozzle stability.

Here, a simplified dosimetric analysis is presented to demonstrate the concept of LIGHT using a single treatment field and a digital XCAT phantom (Segars *et al* 2010), with the 3DRM treatment assumed to be static and delivered to the reference phase. While this is justified considering the presented accuracy of the tracking system with the simplified experimental geometry, the true dosimetric benefits of LIGHT can only be determined with more realistic geometries. The tracking accuracy shown here leaves the positioning errors in the XCAT phantom well below the size of a CT voxel, and hence there is no difference to an ideally gated scenario. However, simulating the full LIGHT process on the XCAT, i.e. generating an ion radiograph for every phase of the XCAT 4DCT, with the triggering method used in experiment, would still yield the correct phase of the 4DCT for the trigger, as this would simply present the smallest difference to the desired tumour position. In reality, breathing patterns are highly irregular and the images would be subject to different motion artefacts, which would influence the triggering. It has been shown that particle radiography simulations using the XCAT phantom can also present highly idealised results compared to reality, given the complex nature of light collection (Simard *et al* 2024). Therefore full evaluation of dosimetric benefits must be experimentally led and should consider other state-of-the-art motion mitigation strategies, such as presented in Steinsberger (2025). In addition, dose calculation based on Monte Carlo simulations would provide a more accurate representation of the 3DRM dose compared to the analytical approximation done here Simeonov *et al* (2017). Based on the promising experiments, a larger patient cohort simulation study to judge the benefits of LIGHT is justified and will be performed in the future.

Nevertheless, the results presented here indicate promising potential of ion-fluoroscopy guided heavy ion therapy. Ultra-fast treatment delivery not only has the potential to improve the dose to the patient, by minimizing motion during delivery, it would also benefit overall patient throughput, by reducing the treatment duration. However, if the tumour is not at the planned position during delivery, a substantial dose could be transferred to healthy tissue. Since the 3DRM cannot be adapted in real time, there is no option to actively adapt the treatment to the tumour position changes like in 4D-synchronized delivery. A gated delivery is the only possible solution. Since LIGHT is by design beams-eye-view, it enables to automatically detect the alignment without registration uncertainties. Moreover, it holds information on the WET of the patient, possibly enabling to infer the range alignment of the 3DRM as well. In addition, 3DRM have been named the most promising solution to achieve ultra-high dose-rate treatments with particle therapy (Jolly *et al* 2020). The high doses and short treatment durations of FLASH therapy in particular make image guidance mandatory. The LIGHT concept would therefore be particularly suited also for possible future FLASH treatments.

## 6. Conclusion

This work introduced a new concept, LIGHT: lung tumour ion-fluoroscopy guided heavy ion therapy. The concept combines recent advances in dynamic ion radiography detectors with the promising treatment option of 3DRM, which permit ultra-fast treatment delivery. Ion-radiography guidance can in principle provide the information for when the tumour is located at the position planned for the 3DRM, permitting safe, ultra-fast treatment delivery. This proof-of-concept work demonstrated the possibility of real-time tumour tracking with dynamic carbon ion radiographs, at a 5 Hz frame rate. In a simplified geometry, the real-time reconstructed tumour position was used to dynamically trigger a treatment, capturing the tumour during free-breathing with a quasi-static dose distribution. A planning study on a digital anthropomorphic phantom showed *in-situ* the potential benefit of this approach over 4DITV treatments, even with rescanning as mitigation strategy. Overall, this work highlights promising potential of LIGHT for future 3DRM treatments of lung tumours. Future work will seek to fully demonstrate driving in the 3DRM using the imaging trigger, test tracking capabilities in more realistic geometry and investigate incorporating WET sensitivity to enable 3D tumour position monitoring.

## Figures and Tables

**Figure 9.**
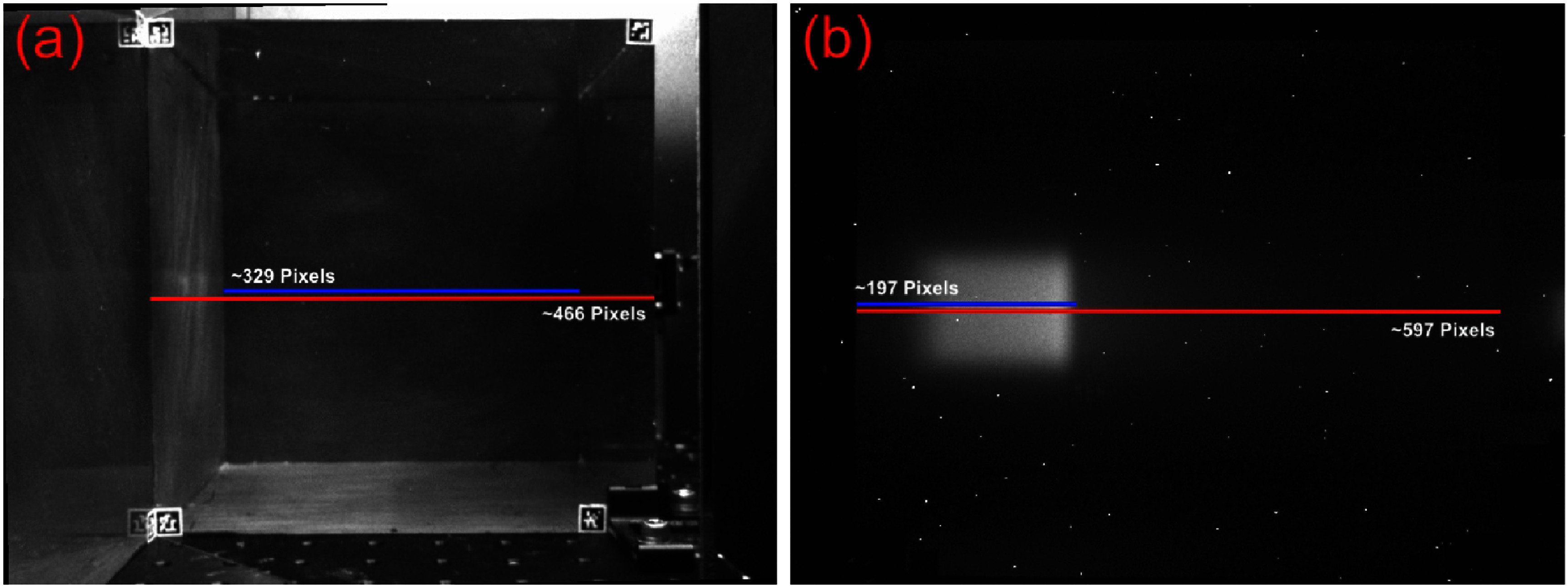
Pixel calibration showing (a) difference between the number of pixels for the front and back faces of scintillator and in the distal view (b) the number of pixels corresponding to the plan depth and full scintillator depth as seen in the lateral view camera.

## Data Availability

The data cannot be made publicly available upon publication because they are not available in a format that is sufficiently accessible or reusable by other researchers. The data that support the findings of this study are available upon reasonable request from the authors.

## Appendix

### A.1. Reproducibility of 3DRM positioning

In order to analyse the reproducibility of a fast automatic 3DRM positioning, a pneumatic-driven target shifter was used to move the 3DRM in and out of the treatment field multiple times. The target shifter is usually used as the range shifter system for GSI’s Cave M (Lis *et al* 2021). While it is not optimized for speed, it enabled to move the 3DRM over a distance of 25 cm in about 1 s, which is close to the desired range for LIGHT, where the envision 3DRM positioning is envisioned to about two to four times as fast. The 3DRM was placed close to the beam nozzle in one of the treatment rooms at MIT, where a horizontal beamline is available. The 3DRM was mounted inside the target shifter by a custom designed 3D-printed holder. A PTW PeakFinder (PTW, Freiburg, GER), which comprises two ICs at either end of an adjustable water column, was placed at isocentre for depth dose measurements. The setup is shown in figure 8(a). The 3DRM was first positioned manually, using feeler gauges to align it as well as possible with the in-room laser system. A reference depth-dose curve was acquired after which the positioning was automatically repeated using the target shifter pneumatic system, recording depth-dose profiles each time. Figure 8(b) shows a comparison of the acquired depth-dose profiles. It should be noted that the RM used in this work is strictly speaking a 2D RM rather than a patient-specific 3DRM and was designed for a homogeneous biological dose, with a slight edge enhancement at the distal fall off. The mean difference between the 2 repetitions shown and the reference was 0.3%.

### A.2. Camera perspective correction

To convert the pixel number for the tracked centroid position to a position in real space, the physical pixel size in millimetres for the scintillator must be calculated. While lens distortion is corrected for during image processing, a significant perspective effect remains in the image such that the pixel width for the back and front faces of the scintillator is different, as shown in figure 9(a). By taking the difference in the pixels between the front and back faces, the ratio of the depth of the delivered field to the total depth of the scintillator from a lateral view image (shown in figure 9(b)) and using a linear approximation to calculate the number of pixels corresponding to the scintillator width at the plan depth, the pixel size calculated to be 0.529 mm.
